# Optimizing continuity-of-care opportunities to reduce health risks: shared qualitative perspectives from CJDATS 2 research

**DOI:** 10.1186/1940-0640-10-S1-A46

**Published:** 2015-02-20

**Authors:** Jennifer Pankow, Yang Yang, Kevin Knight, Wayne EK Lehman

**Affiliations:** 1 Institute of Behavioral Research, Texas Christian University, Fort Worth, TX, 76129, USA; 2 Department of Psychology, University of Louisiana at Lafayette, Lafayette, LA, 70504, USA

## 

Current Texas Christian University (TCU) research focuses on reducing risk for relapse and other health-related behaviors associated with HIV, HBV, and HCV. The *WaySafe* curriculum was developed for prisoners in the last phase of their substance abuse treatment before transitioning back to the community, and was designed to improve decisionmaking and recognizing and planning for risky situations. A critical next step is to shift the setting from incarceration to a community setting with probationers during the high-risk transition period after release from prison or completion of residential or intensive outpatient substance abuse treatment. Literature supports the importance of prevention programs [1] as part of the continuity-of-care for offenders and probationers in addressing some service gaps (in the continuum), which are widely recognized to reduce the likelihood of successful client outcomes [2,3].

The purpose of this presentation is to provide evidence from the recently completed second phase of the Criminal Justice Drug Abuse Treatment Studies (CJDATS 2), with insight from key stakeholders that informs the relevance of continuity-of-care and the significant role that community corrections and community-based health-care agencies fulfill in transitioning probationers from incarceration to home. These qualitative results come from two protocols conducted as part of CJDATS 2: HIV Services and Treatment Implementation in Corrections (HIV-STIC) and Medication-Assisted Treatment in Community Corrections Environments (MATICCE). The organizational-level findings reflect the opinions and views of criminal justice personnel and health-care providers—agencies in partnership with TCU in HIV-STIC and MATICCE. In CJDATS 2, stakeholders participated in an implementation research initiative, utilizing a change team strategy to identify and implement improvements to service delivery. In both HIV-STIC and MATICCE, the services delivery continuum incorporated elements of prison-based services and transition to community-based services in their respective areas. Qualitative results highlight the important contributions of community correction agencies, their direct contact to probationers, and their ability to facilitate linkages to service providers. Findings are discussed, emphasizing the importance of staff commitment and interorganizational relationships in continuity-of-care from prison to community as essential components to optimizing services to probationers.
